# Tuberculosis infection in an urban homeless population

**DOI:** 10.1017/S0950268826101629

**Published:** 2026-05-25

**Authors:** David Moynan, Sean Donohue, Aisling Fitzgerald, Helina Alemayehu, Elizabeth Groarke, Aoife Mooney, Fiona Gaffney, Kelley Ann Brandon, Karen Jones, Niall Conlon, Cliona Ni Cheallaigh, Niamh Allen

**Affiliations:** 1 Department of Infectious Diseases, Mater Misericordiae University Hospital, Dublin, Ireland; 2 School of Medicine, University College Dublin, Ireland; 3 Genitourinary Infectious Diseases Department, St James’s Hospital, Dublin, Ireland; 4 Department of Immunology, LabMed Directorate, St James’s Hospital, Dublin, Ireland; 5 School of Medicine, Trinity College Dublin, Dublin, Ireland

**Keywords:** tuberculosis, LTBI, latent tuberculosis infection, inclusion health, homelessness, IGRA, interferon gamma release assay

## Abstract

People experiencing homelessness (PEH) are at increased risk of tuberculosis (TB) infection and progression to TB disease. Data on TB infection prevalence among hospitalised PEH in low-incidence countries remain limited. This study aimed to estimate TB infection prevalence and describe associated demographic and clinical characteristics among PEH admitted to an urban tertiary hospital in Dublin, Ireland. A prevalence study was conducted between November 2023 and March 2025 using an opt-out screening model. Hospitalised PEH were offered interferon gamma release assay (IGRA) testing on admission. Demographic and clinical data were collected, and positive IGRA results were assessed clinically and radiographically to exclude active TB disease. Estimated lifetime risk of progression to TB disease was calculated using a validated risk-modelling algorithm.Among 118 PEH screened, 13 (11%) were IGRA-positive and 3 (2.5%) had indeterminate results. The cohort was predominantly male (77%) with a mean age of 46 years. Non–Irish-born individuals had higher IGRA positivity than Irish-born participants (23.8% vs 8.3%, p = .054). Substance and alcohol use disorders were common, over half had previous incarceration, and one-third were sleeping rough at admission. Among IGRA-positive individuals, the mean estimated lifetime risk of progression to TB disease was 6.96% (SD 3.3). These findings support strengthened, integrated TB prevention strategies for PEH.

## Introduction

Tuberculosis (TB) remains a major global health concern, even in low-incidence countries like Ireland. The World Health Organisation (WHO) recommends that low-incidence countries (<10 cases per 100,000 population) move toward TB elimination, defined as fewer than 1 case per million annually [1, 2]. Achieving this goal necessitates identifying and managing TB infection in high-risk populations to prevent TB disease [2].

People experiencing homelessness (PEH) have higher TB infection rates and a greater risk of progression to active TB disease [3], driven by factors such as congregate living, poor nutrition, substance use disorders and limited healthcare access. The prevalence of TB infection in PEH varies by setting, but studies have reported rates ranging from 16.5% in London to 36.7% in Poland [4, 5].

In Ireland, a country with an estimated TB infection prevalence of 3.5%, addressing TB among marginalised populations is essential to elimination efforts [1]. The WHO recommends systematic screening for TB infection in populations such as prisoners, people who inject drugs (PWID), migrants from high-TB burden countries and PEH [2]. These groups are often overlooked in health surveillance systems, contributing to delays in diagnosis and increasing the risk of ongoing transmission where disease is present.

This study was conducted at an inner-city tertiary hospital with a specialised inclusion-health service for PEH. The inclusion-health service is a multidisciplinary team that provides inpatient care to PEH, recognising the unique clinical needs of this patient cohort to help combat existent health inequalities. This study aimed to estimate the prevalence of TB infection and describe the demographic and clinical risk factors among the cohort.

## Methods

A prevalence study was conducted from November 2023 to March 2025. All hospitalised inpatients identified by the inclusion-health team as currently homeless – either rough sleeping or residing in hostel accommodation – were eligible for inclusion unless they actively opted out. TB infection screening was offered using an opt-out model, in which eligible patients were informed about the study and the testing process. People living with HIV (PLWH) were excluded as they were screened through separate TB infection protocols.

An interferon gamma release assay (IGRA) was performed at hospital admission on subject samples using standardised protocols in an accredited laboratory (QuantiFERON-TB Gold [6]). Demographic and clinical data were collected, including age, sex, nationality, incarceration history, living situation at time of admission (homeless hostel or ‘sleeping rough’ on the streets), alcohol and substance use, smoking, BCG vaccination and TB exposure history. TB exposure was assessed through patient self-report, defined as any lifetime contact with confirmed or suspected TB cases.

Positive results were reviewed by an infectious diseases clinician to ensure they did not reflect TB disease, based on clinical presentation, physical examination and radiography. We calculated each patient’s estimated cumulative risk of TB reactivation up to age 80, based on a validated model by *Menzies et al.* [7]. This model calculates an individual’s annual risk of developing active disease by combining the baseline annual incidence observed in healthy populations with the patient’s specific relative risk based on clinical and radiographic factors. The resulting annual disease risk is then multiplied by the positive predictive value and by the patient’s expected years of survival to yield an estimated cumulative lifetime risk of TB disease [7].

Power calculations indicated that a sample size of 115 was adequate to estimate TB infection prevalence at 8% with 95% confidence and a +/−5% precision. The study was powered to estimate TB infection prevalence, not to detect associations between risk factors, and results of subgroup analyses should therefore be interpreted cautiously. Statistical analysis was performed using STATA/SE v18.5. Categorical variables were analysed using Fisher’s test and continuous variables using *t*-test.

The study was approved by the Research and Innovation Office as a quality improvement initiative and conducted in accordance with the Declaration of Helsinki with all patients verbally consented for testing.

## Results

Of the 118 patients screened, 13 (11%) had a positive IGRA, deemed clinically consistent with TB infection. Three patients (2.5%) had an indeterminate result. The cohort was predominately male (77%) with a mean age of 46 years (standard deviation (SD) 10.3 years). The majority of the study cohort was Irish-born (97/118; 82.2%) with full demographics noted in Table 1. The proportion of IGRA positivity, however, was three times higher among non-Irish individuals compared to their Irish counterparts (5/21; 23.8% vs. 8/97; 8.3% *p* = .054). There were high proportions of substance and alcohol use disorders, over half had prior incarceration and one-third were unsheltered (‘rough sleeping’) at time of admission. While this study was not powered to examine these traditional risk factors, there was no significant association found between IGRA positivity and these variables (Table 2).

**Table 1.** Summary statistics of screened patients Table 1. long description.

Summary statistics	
Total screened	118 (100%)
Total positive	13 (11%)
Total indeterminate	3 (2.5%)
Male	91 (77%)
Age	46 years (SD 10.3)
18–29 years	6 (5%)
30–49 years	70 (59%)
50–64 years	33 (28%)
65 + years	9 (8%)
Nationality	
Ireland	97 (82.2%)
United Kingdom	3 (2.5%)
Other European	9 (7.6%)
*Poland*	5
*Latvia*	2
*Romania*	1
*Hungary*	1
Sub-Saharan Africa	4 (3.4%)
*Nigeria*	2
*Malawi*	1
*Somalia*	1
Asia	3 (2.5%)
*Syria*	1
*Mongolia*	1
*Pakistan*	1
Americas	2 (1.8%)
*USA*	1
*Bra*zil	1
Substance use	
Alcohol use disorder	77 (65%)
Active PWID	23 (20%)
Former PWID	34 (29%)
Accommodation	
Living in hostel	76 (65%)
‘Rough sleeping’	42 (35%)
Previous incarceration	69 (58.5%)
TB status	
Prior TB exposure	11 (9.7 %)
History of TB	2 (1.7%)
BCG vaccination	47 (50.5%)

*Note:* PWID: people who inject drugs, TB: tuberculosis, BCG: Bacille Calmette-Guérin.

**Table 2.** Association between demographic and clinical factors and IGRA positivity among PEH Table 2. long description.

	IGRA positive N (%)	IGRA negative N (%)	*p*-value
Country of origin			
Ireland	8 (6.8%)	89 (75.4%)	0.054
Other	5 (4.2%)	16 (13.6%)	
Alcohol use disorder			
Yes	9 (7.6%)	68 (57.6%)	1.000
No	4 (3.4%)	37 (31.4%)	
PWID			
Yes	5 (4.2%)	52 (44.1%)	0.561
No	8 (6.8%)	53 (44.9%)	
Rough sleeping			
Yes	5 (4.2%)	37 (31.4%)	1.000
No	8 (6.8%)	68 (57.6%)	
Previous incarceration			
Yes	6 (5.1%)	63 (53.4%)	0.381
No	7 (5.9%)	42 (35.6%)	

*Note:* Statistical comparisons were performed using Fisher’s exact test for categorical variables.

Risk modelling (Table 3) using existing literature [7] was performed on the 13 IGRA-positive patients. The cumulative lifetime risk of progression to TB disease varied by age, country of origin, smoking status, BCG vaccination status and previous self-reported TB exposure (mean risk 6.96%, SD 3.3; 95% confidence interval (CI) 4.44–9.49).

**Table 3.** IGRA-positive cohort with risk modelling of TB disease [7] Table 3. long description.

Patient Number	Age	Sex	BCG	TB exposure	Previous incarceration	Nationality	TB Disease Risk (80 Years)
1	48	Male	Yes	No	Yes	Ireland	7.84%
2	54	Male	No	No	No	Ireland	6.37%
3	57	Male	Yes	No	No	Ireland	7.84%
4	48	Female	No	No	No	Ireland	7.84%
5	54	Male	Yes	No	Yes	Ireland	5.64%
6	52	Male	Yes	No	No	Pakistan	14.54%
7	45	Male	Yes	Yes	Yes	Ireland	8.57%
8	48	Male	No	No	No	Malawi	3.14%
9	69	Male	No	No	No	Ireland	2.7%
10	44	Male	No	No	No	Poland	8.82%
11	68	Male	Unknown	No	No	Ireland	2.94%
12	36	Male	Yes	No	No	Brazil	10.78%
13	59	Male	Yes	No	Yes	Romania	2.06%

## Discussion

This study demonstrates a notable period prevalence of TB infection among hospitalised PEH in a tertiary hospital in Dublin, with 11% of subjects testing IGRA-positive. This figure is nearly three times the estimated prevalence of the general Irish population and is consistent with international data from comparable urban settings [1, [4], 5].

Country of origin showed a trend toward an association with IGRA positivity, with non-Irish subjects demonstrating a higher prevalence of TB infection compared to Irish-born individuals (23.8% vs. 8.2%, Fisher’s exact test, *p* = .054). In contrast, traditional TB infection risk factors such as prior incarceration, alcohol use disorder and substance use were not significantly associated with infection [4]. However, this study was not powered to detect small differences between subgroups, and these results should therefore be interpreted with caution. Taken together, the findings suggest that while screening among migrants remains important and consistent with current recommendations, a broad-based screening approach may be more appropriate for PEH, given the uniformly elevated risk across this population [9]

The mean cumulative risk of progressing from TB infection to active TB disease was higher among non–Irish-born individuals compared with Irish-born subjects (7.9% vs. 6.2%), although this difference was not statistically significant (*p* = .46). While TB infection screening efforts frequently prioritise foreign-born populations due to the higher background prevalence of TB in some regions, these findings highlight that homelessness itself confers a substantial risk of TB infection. In Ireland, several structured approaches to TB infection screening are already in place, including occupational health assessments for healthcare workers and screening prior to the initiation of biologic or other immunosuppressive therapies [9]. National guidance has also recommended the expansion of TB infection screening to additional high-risk populations, including people who are incarcerated within the Irish Prison Service. This recommendation was informed by a similar study that reported a TB infection prevalence of approximately 9% in a comparable high-risk incarcerated group, and which likewise found that individuals born outside Ireland were twice as likely to have TB infection as those born in Ireland [10].

The use of IGRA testing enables specific and reliable identification of TB infection, even in individuals with prior BCG vaccination [5] although significant challenges remain around treatment initiation and completion. Following on from the completion of this study, all positive cases were reviewed for treatment consideration. Two IGRA-positive patients of the 11 that tested positive were clinically suitable to commence treatment, factoring in active addiction, unavoidable drug-drug interactions and established chronic liver disease, factors which precluded therapy for many. Of these two patients, one was discontinued early, highlighting substantial structural and psychological barriers in maintaining healthcare engagement among PEH undergoing TB infection treatment. However, while TB screening should be linked to treatment, identifying TB infection remains important, even in the absence of treatment initiation, as it allows for targeted monitoring of those at higher risk as part of a broader TB care pathway.

TB management in the context of addiction and housing instability requires an integrated approach. Effective strategies include co-locating TB care with addiction and housing services, using mobile units and outreach teams and offering short-course, once-weekly TB infection regimens to reduce treatment burden without compromising efficacy [8]. Directly observed therapy can support adherence and treatment completion, but screening and treating high-risk groups requires sufficient funding, staffing and long-term planning to ensure effectiveness and sustainability.

This single-centre prevalence study has several limitations. The sample size was modest and powered to estimate TB infection prevalence over the study period rather than detect associations between risk factors, limiting subgroup analysis. Recruitment from a single tertiary hospital with an inclusion-health service may introduce selection bias and restrict generalisability to other settings or homeless populations not engaged in inpatient care, while self-reporting previous TB exposure is subject to recall and reporting bias. The exclusion of PLWH may have led to underestimation of the true TB infection burden. As a cross-sectional study, causal relationships between potential risk factors and TB infection cannot be inferred.

Nonetheless, the findings provide valuable local evidence to inform national TB strategies and highlight the need for investment in inclusion-health and flexible, patient-centred models of TB infection care.

## Data Availability

Due to ethical and privacy considerations, the data that support the findings of this study are available from the corresponding author on reasonable request and with permission from the hospital’s Research and Innovation Office.

## [Table 1] Long description

Starting from the top, the table lists summary statistics. Total screened: 118 (100 percent). Total positive: 13 (11 percent). Total indeterminate: 3 (2.5 percent). Male: 91 (77 percent). Age: 46 years, standard deviation 10.3. Age groups: 18 to 29 years, 6 (5 percent); 30 to 49 years, 70 (59 percent); 50 to 64 years, 33 (28 percent); 65 plus years, 9 (8 percent). Nationality section: Ireland, 97 (82.2 percent); United Kingdom, 3 (2.5 percent); Other European, 9 (7.6 percent), with Poland 5, Latvia 2, Romania 1, Hungary 1. Sub-Saharan Africa, 4 (3.4 percent), with Nigeria 2, Malawi 1, Somalia 1. Asia, 3 (2.5 percent), with Syria 1, Mongolia 1, Pakistan 1. Americas, 2 (1.8 percent), with U S A 1, Brazil 1. Substance use: Alcohol use disorder, 77 (65 percent); Active P W I D, 23 (20 percent); Former P W I D, 34 (29 percent). Accommodation: Living in hostel, 76 (65 percent); Rough sleeping, 42 (35 percent). Previous incarceration: 69 (58.5 percent). T B status: Prior T B exposure, 11 (9.7 percent); History of T B, 2 (1.7 percent); B C G vaccination, 47 (50.5 percent). Footnote: P W I D is people who inject drugs, T B is tuberculosis, B C G is Bacille Calmette-Guerin.

Navigate back to [Table 1].

## [Table 2] Long description

Starting from the top row, the table lists demographic and clinical factors in the first column: country of origin, alcohol use disorder, P W I D, rough sleeping, and previous incarceration. For country of origin, Ireland shows 8 IGRA positive (6.8 percent), 89 IGRA negative (75.4 percent), p-value 0.054; Other shows 5 IGRA positive (4.2 percent), 16 IGRA negative (13.6 percent), no p-value. For alcohol use disorder, Yes has 9 IGRA positive (7.6 percent), 68 IGRA negative (57.6 percent), p-value 1.000; No has 4 IGRA positive (3.4 percent), 37 IGRA negative (31.4 percent), no p-value. For P W I D, Yes has 5 IGRA positive (4.2 percent), 52 IGRA negative (44.1 percent), p-value 0.561; No has 8 IGRA positive (6.8 percent), 53 IGRA negative (44.9 percent), no p-value. For rough sleeping, Yes has 5 IGRA positive (4.2 percent), 37 IGRA negative (31.4 percent), p-value 1.000; No has 8 IGRA positive (6.8 percent), 68 IGRA negative (57.6 percent), no p-value. For previous incarceration, Yes has 6 IGRA positive (5.1 percent), 63 IGRA negative (53.4 percent), p-value 0.381; No has 7 IGRA positive (5.9 percent), 42 IGRA negative (35.6 percent), no p-value. Statistical comparisons were performed using Fisher’s exact test for categorical variables.

Navigate back to [Table 2].

## [Table 3] Long description

The header row contains Patient Number, Age, Sex, B C G status, TB exposure, Previous incarceration, Nationality, and TB Disease Risk at 80 Years. Patient entries are as follows, from top to bottom: 1, age 48, male, B C G yes, TB exposure no, previous incarceration yes, nationality Ireland, TB disease risk 7.84 percent. 2, age 54, male, B C G no, TB exposure no, previous incarceration no, nationality Ireland, TB disease risk 6.37 percent. 3, age 57, male, B C G yes, TB exposure no, previous incarceration no, nationality Ireland, TB disease risk 7.84 percent. 4, age 48, female, B C G no, TB exposure no, previous incarceration no, nationality Ireland, TB disease risk 7.84 percent. 5, age 54, male, B C G yes, TB exposure no, previous incarceration yes, nationality Ireland, TB disease risk 5.64 percent. 6, age 52, male, B C G yes, TB exposure no, previous incarceration no, nationality Pakistan, TB disease risk 14.54 percent. 7, age 45, male, B C G yes, TB exposure yes, previous incarceration yes, nationality Ireland, TB disease risk 8.57 percent. 8, age 48, male, B C G no, TB exposure no, previous incarceration no, nationality Malawi, TB disease risk 3.14 percent. 9, age 69, male, B C G no, TB exposure no, previous incarceration no, nationality Ireland, TB disease risk 2.7 percent. 10, age 44, male, B C G no, TB exposure no, previous incarceration no, nationality Poland, TB disease risk 8.82 percent. 11, age 68, male, B C G unknown, TB exposure no, previous incarceration no, nationality Ireland, TB disease risk 2.94 percent. 12, age 36, male, B C G yes, TB exposure no, previous incarceration no, nationality Brazil, TB disease risk 10.78 percent. 13, age 59, male, B C G yes, TB exposure no, previous incarceration yes, nationality Romania, TB disease risk 2.06 percent. The highest TB disease risk is 14.54 percent for a male from Pakistan, and the lowest is 2.06 percent for a male from Romania. Most patients are male and from Ireland, with varied B C G status and previous incarceration history.

Navigate back to [Table 3].
